# On the Interplay Between the Medicine of Hildegard of Bingen and Modern Medicine: The Role of Estrogen Receptor as an Example of Biodynamic Interface for Studying the Chronic Disease's Complexity

**DOI:** 10.3389/fnins.2022.745138

**Published:** 2022-05-25

**Authors:** Sabrina Melino, Elisabetta Mormone

**Affiliations:** ^1^Research Unit of Philosophy of Science and Human Development, Faculty of Science and Technology for Humans and the Environment, University Campus Bio-Medico of Rome, Rome, Italy; ^2^Fondazione IRCCS Casa Sollievo della Sofferenza, Institute for Stem-Cell Biology, Regenerative Medicine and Innovative Therapies, Foggia, Italy

**Keywords:** behavior, biodynamic interfaces, epigenetic, estrogen, Hildegard of Bingen, HPA axis, sex, temperament

## Abstract

**Introduction:**

Hildegard of Bingen (1098-1179) interpreted the origins of chronic disease highlighting and anticipating, although only in a limited fashion, the importance that complex interactions among numerous genetic, internal milieu and external environmental factors have in determining the disease phenotype. Today, we recognize those factors, capable of mediating the transmission of messages between human body and environment and *vice versa*, as biodynamic interfaces.

**Aim:**

We analyzed, in the light of modern scientific evidence, Hildegard of Bingen's medical approach and her original humoral theory in order to identify possible insights included in her medicine that could be referred to in the context of modern evidence-based medicine. In particular, the abbess's humoral theory suggests the identification of biodynamic interfaces with sex hormones and their receptors.

**Findings:**

We found that the Hildegardian holistic vision of the organism-environment relationship can actually represent a visionary approach to modern endocrinology and that sex hormones, in particular estrogens, could represent an example of a biodynamic interface. Estrogen receptors are found in regions of the brain involved in emotional and cognitive regulation, controlling the molecular mechanism of brain function. Estrogen receptors are involved in the regulation of the hypothalamic-pituitary-adrenal axis and in the epigenetic regulation of responses to physiological, social, and hormonal stimuli. Furthermore, estrogen affects gene methylation on its own and related receptor promoters in discrete regions of the developing brain. This scenario was strikingly perceived by the abbess in the XIIth century, and depicted as a complex interplay among different humors and flegmata that she recognized to be sex specific and environmentally regulated.

**Viewpoint:**

Considering the function played by hormones, analyzed through the last scientific evidence, and scientific literature on biodynamic interfaces, we could suggest Hildegardian insights and theories as the first attempt to describe the modern holistic, sex-based medicine.

**Conclusion:**

Hildegard anticipated a concept of pathogenesis that sees a central role for endocrinology in sex-specific disease. Furthermore, estrogens and estrogen receptors could represent a good example of molecular interfaces capable of modulating the interaction between the organism internal milieu and the environmental factors.

## Introduction

**Figure 1:**
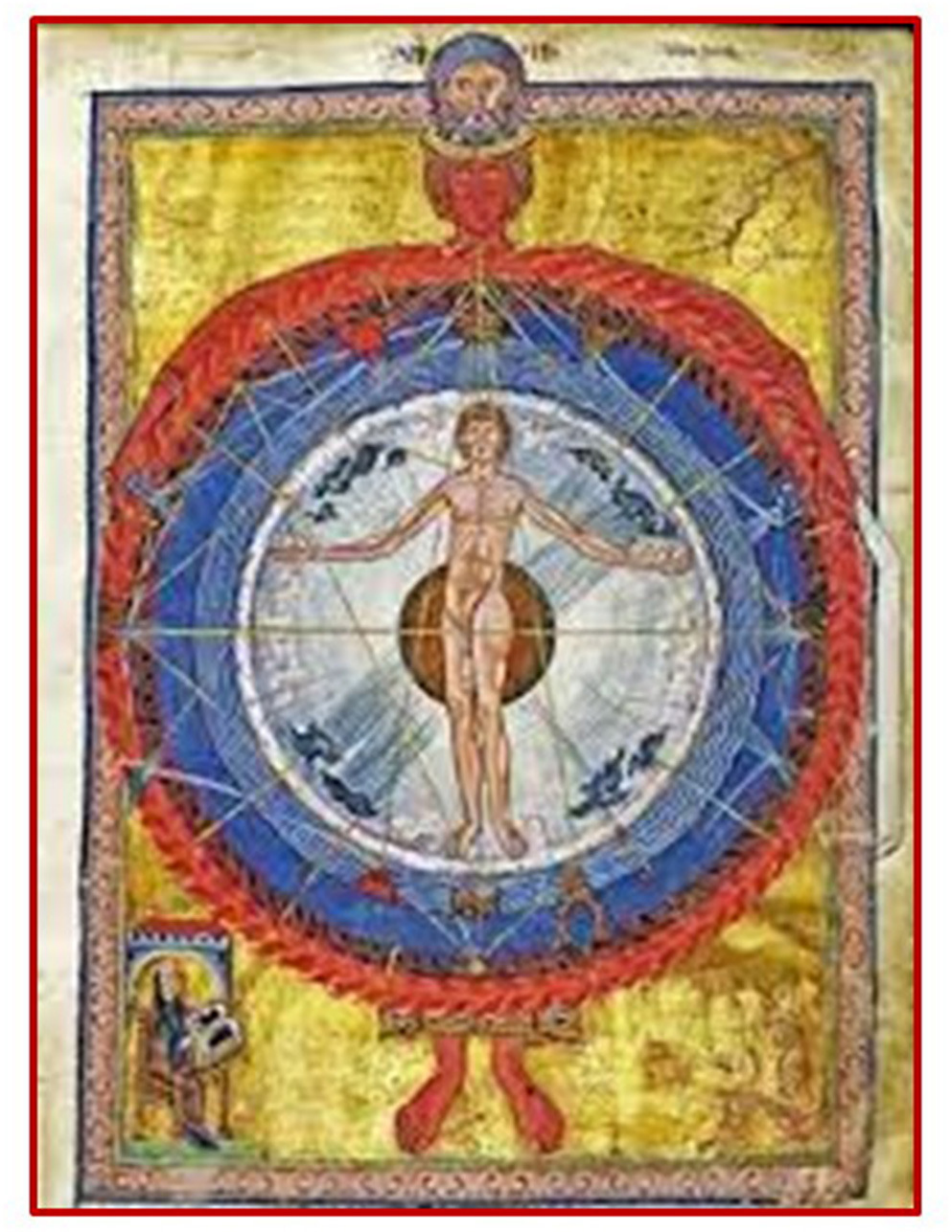
Liber divinorum operum, f 9r., sec. XIII, Biblioteca Statale di Lucca, ms. 1942: it represents the close relationship between human beings and the universe, between macrocosm and microcosm. The wheel delimited by the embrace of the Spirit fire represents all of the creation made up of the four elements: air, water, earth, and fire. The four elements are closely integrated and interpenetrated with one another. At the center of the wheel appears the human being: the winds' blows and the cosmic rays converge on it to demonstrate the close relationship of interdependence between visible and invisible and between macrocosm and microcosm. The bottom left is Hildegard von Bingen.

Although therapeutic options for several diseases have significantly been improved over the last few years, the “WHO's 2019 Global Health Estimates report” reveals trends over the former two decades, in mortality and morbidity, caused by diseases and injuries. This highlights the need for an intensified global focus on preventing and treating cardiovascular diseases, cancer, diabetes, and chronic respiratory diseases, as well as tackling injuries. According to WHO in 2019, non-communicable diseases made up 7 of the world's top 10 causes of death. Genomics, at the heart of both personalized and precision medicine, has proved ineffective in treating many chronic diseases. The cause depends on various factors, the same “causal” factor often supporting very different pathogenic phenotypes. Furthermore, for three main reasons, a purely genetic approach is unlikely to be a solution to common diseases. The first reason is the great importance of the environment circumstances in determining health, the second is the great complexity of gene/gene, gene/environment interactions, and the third one is the high degree of individual variability (Bizzarri, 2020). The complexity of chronic diseases is due to the combination of various factors. In fact, the effect of the environment is not only expressed through toxic and infectious agents but also through other factors, such as social, educational, political, and economic conditions. Type 2 diabetes, obesity, cancer, autoimmune forms, stroke, cardiovascular diseases, lung diseases, liver diseases are strongly influenced by social factors that can increase exposure to risk and the susceptibility to contract the disease regardless of whether it is infectious, genetic, metabolic, malignant, or degenerative (Christakis and Fowler, 2007; Calver et al., 2010; Cockerham et al., 2017). These pieces of evidence illustrate the importance of coupling biologic with environmental, social, and policy-based network analysis to construct a more convincing holism for elucidating disease (Greene and Loscalzo, 2017). The complexity of biological systems and organism-environment relationship requires, therefore, a method of study and analysis based on measurable factors that must be inclusive of the changes coming from both systems: from the human organism and from the environment in which it comes into contact with. In agreement with the study of Arora and colleagues (Arora et al., 2020), we would like to identify these measurable factors as biodynamic interfaces, namely, dynamic structures based on natural processes. In their study, they conjecture that complex systems cannot interact directly but do so *via* one or more interfaces that incorporate components capable of responding from both the environment and human physiology but operationally independent, therefore dynamic. From these premises, our questions are: in the personalization of care, it is necessary to follow the reductionist model, which aims to define the specific genetic variation with respect to the variability of the disease or, on the opposite, to use a holistic model that considers the specificity in the relationship between complex systems (human being and environment)? In order to study the mechanism of signal transduction, between the environment and the organism, which, in turn, reflects on the ability to maintain health or to switch to a state of imbalance, which interface should we be looking for? With the aim of finding confirmation of our hypothesis, about the existence of biodynamic interfaces, we began our review dating back to the medieval culture. They had well-understood the profound meaning of the human being-cosmos relationship, in which the cosmos becomes the essential tool for the recognition of human being himself or herself. The abbess Hildegard of Bingen (1098-1179) was able to give an interpretation of the origin of the disease that, today, is reflected, to our opinion, in modern scientific research. We took inspiration from her holistic approach to reviewing in an original way the last scientific works in order to read the disease and the molecular pathways underlying it, in connection to the social, cultural, and natural environment. In describing the humoral theory and giving such an original description of temperament, she provided not only a clear distinction between man and woman, but she highlighted the dependence of the balance of humors with the elements of the cosmos in order to underline the essential link between human beings and the environment (Flanagan, 1996; Moulinier, 2003). Nowadays, epigenetic studies carried out on humans demonstrate how environmental factors, ranging from stress to various chemicals, may alter DNA and, consequently, its expression (Westberry et al., 2011; Sears and Genuis, 2012) and how this epigenetic modulation of DNA differs between the two sexes (Wang et al., 2017). Starting from Hildegard of Bingen's vision and the importance she gives to sex differences, we thought to identify in sex hormones and, in particular, estrogens, a biodynamic interface which intervenes in the transmission of messages between the environment and the organism in both directions. In particular, following her intuition and the importance she attributed to the process of emotions and feelings, we focused on the sensitivity of estrogens with respect to the world of emotions that nourish our relationship life and that modify our internal milieu. Today, we know that estrogens and progesterone act in brain regions, involved in emotional and cognitive regulation (Toffoletto et al., 2014), while the estrogen receptor acts in the stress response (Chen et al., 2008; Weiser and Handa, 2009). We found in Hildegard of Bingen's medicine possible insights, which could be referred as to the scenario that evidence-based medicine depicts in the modern era. This highlights the importance that precision medicine should give to the psychosocial and environmental aspects, which distinguish a given patient from other patients with similar clinical presentations in order to use an individualized model of precision medicine. In particular, we speculated that her eccentric humoral theory suggests the identification of factors, capable of mediating the transmission of messages between the environment and the body and *vice versa*, today called biodynamic interfaces and by her iconized symbolic images, such as the “Egg” and the “Wheel of admirable vision” (Figure 1). Implicitly, following Hildegard of Bingen's footsteps and her description of the origin of the disease, we wanted to give to her medicine and vision of illness a modern interpretation based on the review of the most recent discoveries and knowledge in the fields of endocrinology, molecular biology, and neurobiology. This recognition could be the first example of a scientific revision of Hildegard of Bingen's medical thought, which, in our opinion, could lead to the opening of new lines of research in various fields.

## Hildegard of Bingen'Holistic Approach: a Key to Read the Complexity

### The Complexity of Organism-Environment Relationship

Health is defined by the World Health Organization (WHO) as “a state of complete physical, mental, social, and spiritual well-being, not the mere absence of disease” (from the WHO protocol of constitution, July 22, 1946). All living beings, in order to remain in healthy condition, must maintain the homeostasis, which is a state of dynamic equilibrium of the body that tends to be constantly altered by the frequent changing and, sometimes, unfavorable conditions of the natural and social environment. Health is, therefore, a condition of dynamic equilibrium, in which an individual creates a harmonious relationship with the environment where he or she lives and of which he she is an integral part. By environment, in this case, we mean not only the natural environment, which provides all the elements necessary for “physical” survival (nutrients, water, oxygen) but also the social, economic, political, cultural, and spiritual contexts that provide the elements for “subjective” well being (Puchalski et al., 2014; Vander Weele et al., 2017). The state of health of an individual and, more broadly, of a community or population is, therefore, influenced and determined by the interaction of multiple factors. Considering this dynamism, if we want to attempt to prevent the disease and to study the determinants of health, it is necessary to identify and possibly modify those dynamic factors that act in the organism as biodynamic interfaces (Arora et al., 2020). The interaction between two complex systems, such as human physiology and the environment, with their multilevel organizations, cannot take place directly but requires the intermediation of a biodynamic interface: a structure capable of changing according to the stimuli that it receives both from the physiological and environmental system, assuming distinct and measurable characteristics (Arora et al., 2020). Through the properties of the interface and its modifications, it would be possible to evaluate the impact of the interaction between the environment and human physiology on the origin of disease and health.

### Hildegard's of Bingen Holistic Vision for Reading of the Biological System's Complexity

**Figure 2:**
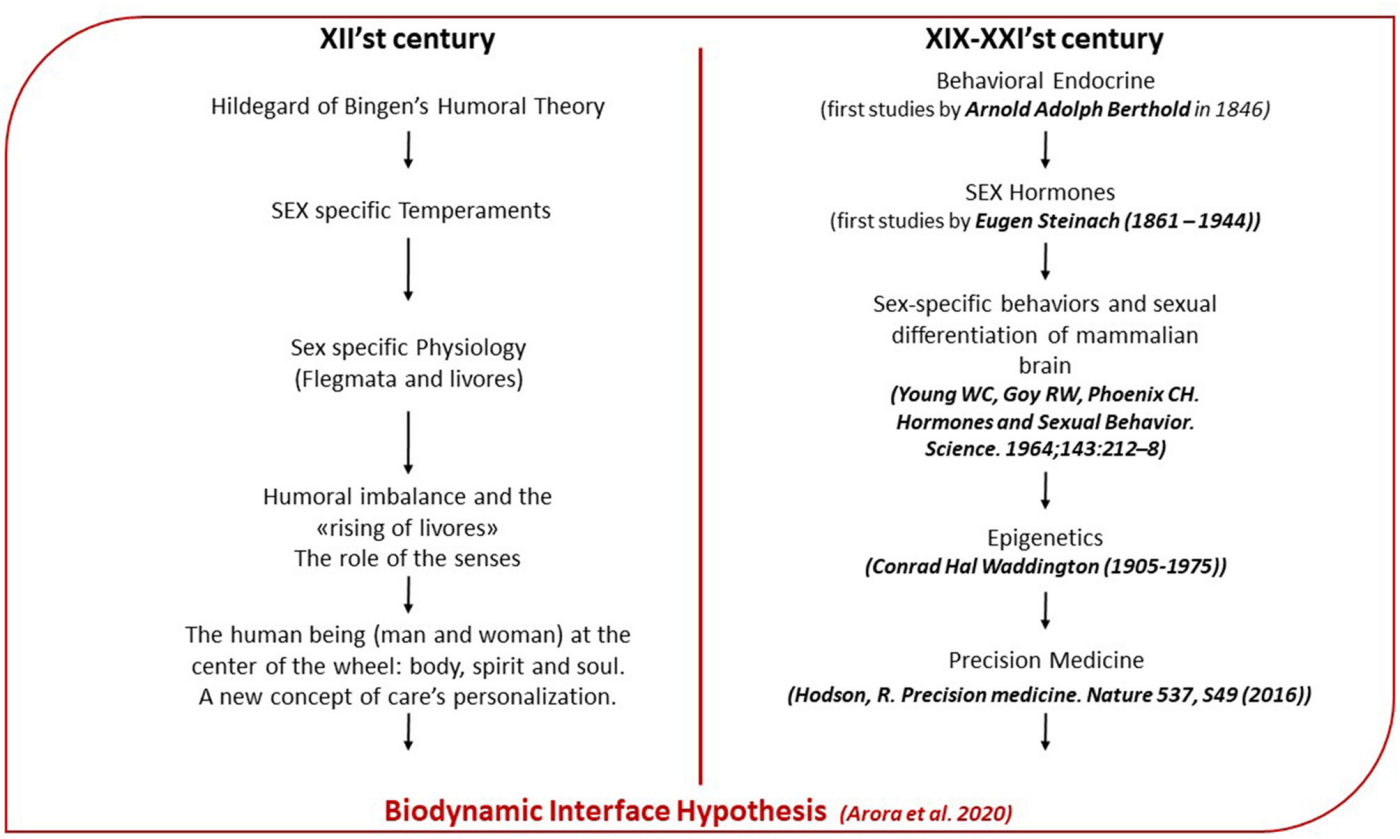
Parallelism between Hildegardian medicine and modern medicine: on the left of the flow chart are summarized main aspects of the Hildegardian medicine. On the right are the reported fields of the modern medicine wherein Hildegardian medicine finds a modern interpretation.

**Figure 3:**
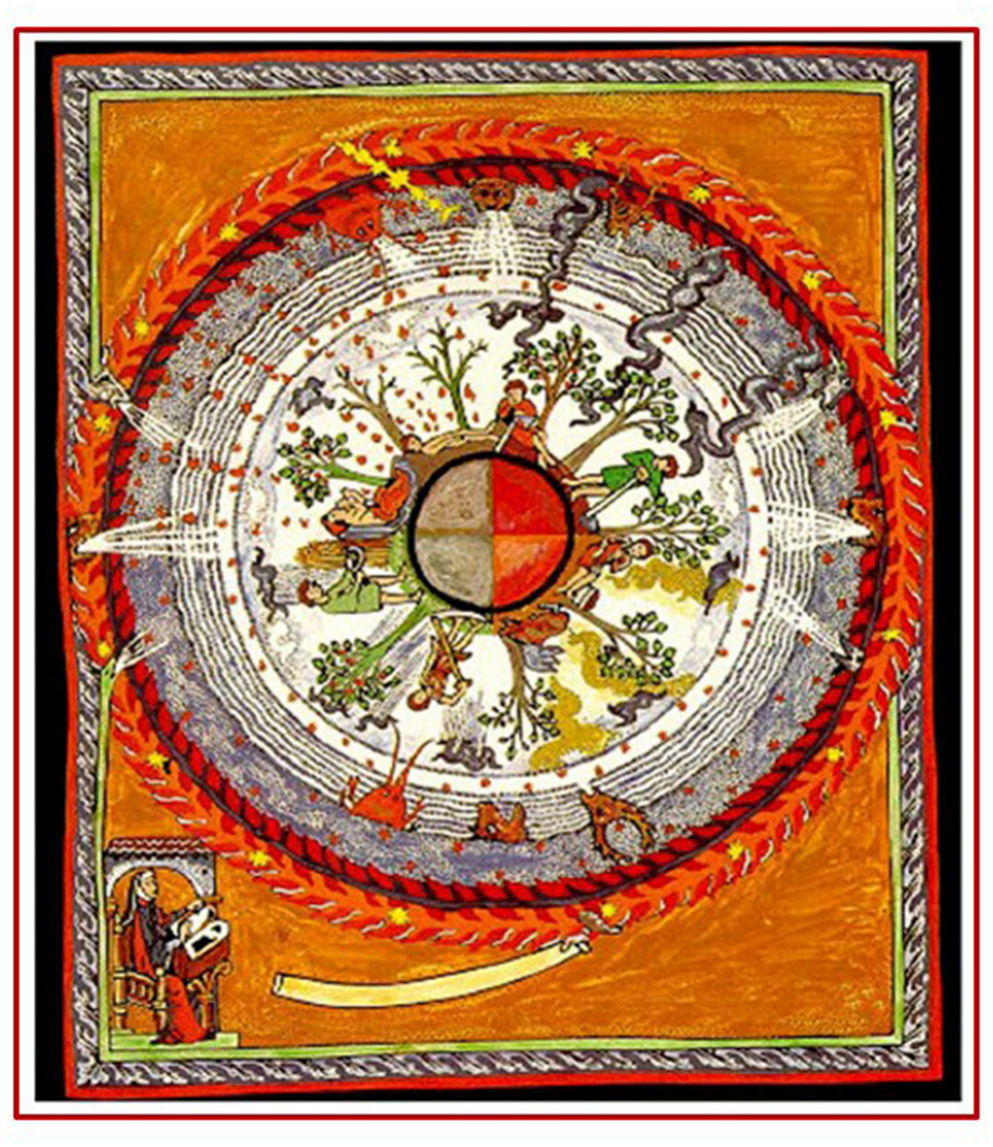
Liber divinorum operum, f 38r., sec. XIII, Biblioteca Statale di Lucca, ms. 1942: In this vision, Hildegard gives importance to the Earth and to the influence that the higher elements exert on it. In particular, the four seasons and the man's work are illustrated. The symbols of animal heads and the breath of winds underline the interdependence between all terrestrial and cosmic phenomena not only from a natural and physical point of view but also from an eschatological one's. Lightning bolts and a smoky fog fall from the firmament on men capable of causing weakness and disease (represented by the suffering human figures) whenever man sins, disobeying the Living Light or whenever the soul and body are not in harmony. The image, therefore, summarizes the Hildegardian vision of the correspondence between human health and salvation.

In order to investigate these interfaces, we decided to carry on our work, following the Hildegard of Bingen thoughts and theories. We thought that the abbess envisioned some concepts of the modern medicine and molecular biology (Figure 2), although using anallegoric and symbolic language, distant from the modern one, used by scientists and physicians, but able to synthesize different concepts in a single word or image. Hildegard of Bingen uniquely emphasized the importance of human health, making it a central topic in all her works, both the more specifically naturalistic and the prophetic ones (Walker-Moskop, 1985). However, for interpreting her language and contextualizing it to modern medicine, we first need to mention the medieval concept of body and disease. The body, in all medieval culture and, specifically, in Hildegard of Bingen, has a meaning that transcends the physiological dimension: It is a space in which pieces of scientific, cultural, and spiritual knowledge merge. For the medieval culture, the concept of interdependence, between microcosm and macrocosm, makes the processes of the human organism inseparable from those of the environment with which human beings relate. The idea of a person is conceptualized as an integral entity in which sensation, emotion, reasoning, and identity are physically connected (Marron, 2014). In Hildegard of Bingen, as in all medieval culture, the link between microcosm and macrocosm is described through the theory of elements and humors, defined in Galen as complexio or a specific balance between the humors, which are formed starting from the four elements: air, water, earth, and fire. However, Hildegard of Bingen developed an original humoral theory, fitting it into the overall cosmology (Flanagan, 1996). It is impossible in the Hildegardian vision to separate the history of human existence from that of the cosmos and studying the principles that regulate human health as independent from the environment in which a human being lives and relates (Figure 3). Therefore, understanding the principles that regulate the balance of the human body means studying the body in its specific interaction with the environment through the involvement of the senses and the processes that Hildegard would attribute to the soul or to the spiritual dimension: intuition, will, understanding (Calef, 1997).

## Hildegard of Bingen's Humoral Theory: the Sex Difference and Temperament Through the Hormones

### Hildegard's Humoral Theory and Sex Difference

**Figure 4:**
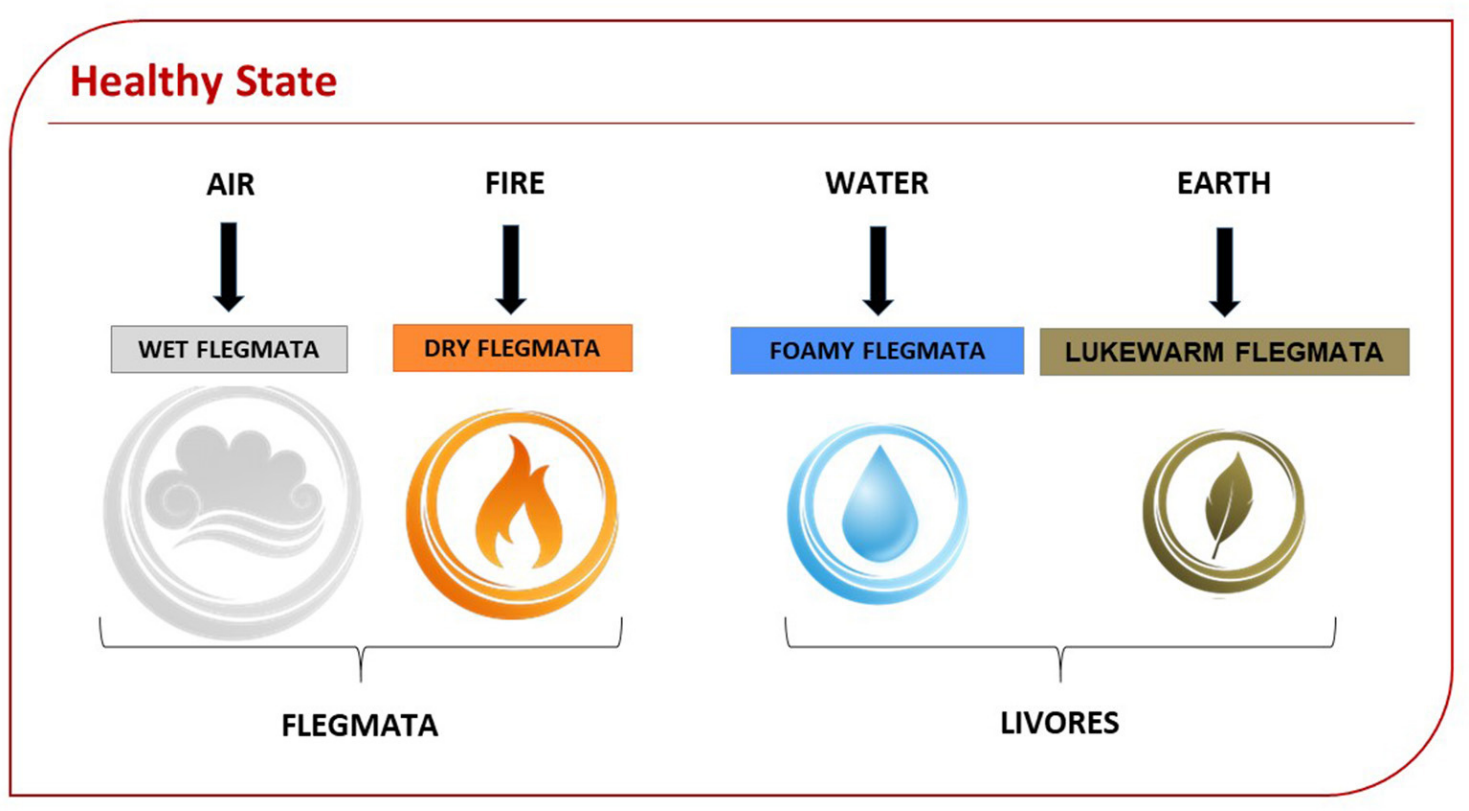
Health state based on Humoral Theory: Humors have decreasing proportions. The first flegmata defines innate temperament. Here is represented the physiology corresponding to the state of health (resilient). The wet flegmata corresponding to the element of air is, for Hildegard, “the nerve's and rationality's way,” confirming that the senses and the thoughts play a central role in balancing humors.

In the Hildegard of Bingen's humoral theory, the elements of the cosmos become four humors and, after the original sin, they are transformed into opposing *flegmata* (the term coined by Hildegard of Bingen), which contain in their nature the predisposition to disease. The human being, the microcosm, is intimately linked to the macrocosm: *his or her humors or flegmata*, says Hildegard of Bingen, *move according to the affinity between their airy component and that of the cosmic winds, which move the universe*. Harmony and health are achieved only if there is a balance between the human being and the cosmos: Each event is reflected in the other, in a perfect circular dynamic, which involves our senses, organs, and minds. Therefore, according to Hildegard, each person is born with a specific proportion between the four humors (dry flegmata, wet flegmata, foamy flegmata, and lukewarm flegmata) (Figure 4). Hildegard defines the two flegmata present in smaller proportions with the term “livores,” giving them the property of “rising” in correspondence with stimuli coming from inside the organism or from the outside. The prevailing flegmata, on the other hand, defines the innate temperament of the individual, in turn distinct in four types: sanguine, melancholic, flegmatic, and bilious. The secondary flegmata or livores can alter the prevailing flegmata and generate an overall imbalance (Figure 2). According to the prevailing flegmata and the proportion between flegmata and livores, Hildegard describes twenty-four different physiologies: six for each prevailing flegmata and, therefore, for each temperament (Sweet, 2006). Each physiology is based on Hildegard's specific reference to character and personality. Temperament is associated with a specific psychophysical balance and defines the prevailing physical characteristics and the psychological, behavioral ones. She introduced a further distinction, according to which temperaments are based on sexual behavior and are distinguished into eight different types, separating man from woman. In distinguishing temperaments on a sex basis, Hildegard of Bingen gave us a unique theory about temperaments, which was not previously attested (Flanagan, 1996). The concept of temperament according to the humoral theory has returned of interest to modern science, which is reconsidering it in the neuropsychiatric field. In fact, modern neuropsychiatry is re-evaluating the neurobiological aspect underlying the humoral theory, because the integrated and multidimensional view of the latter can better interpret the conditions underlying personality disorders (Ross and Margolis, 2018). The humoral theory also relates temperaments to the level of emotional intelligence that, together, with intellectual and social intelligence, define the personality. Considering the importance that Hildegard of Bingen gave to sex differences to define temperaments and interpreting the “rising” of livores as a consequential response to emotional and environmental stimuli, we hypothesized that Hildegard suggested an intermediary role for sex hormones, capable of defining both the organism-environment relationship and the evolution of the pathogenetic process.

### Hormones and Their Role as Mediators of the Relationship Organism Environment

Modern medicine shows us that, in order to maintain the homeostasis, our body activates hormone secretion and turns on our autonomic and central nervous system, helping us to adapt to our daily activities. The “mediators,” like cortisol, adrenalin, the immune system, and metabolism, help us adapt as long as they are turned on, in a balanced way, when we need them and then turned off again when the challenge is over. When this does not happen, they can cause unhealthy changes in the brain and body. This is also the case when the “mediators” are not produced in an orchestrated and balanced manner, for example, too much or too little cortisol in the hypothalamic-pituitary-adrenal (HPA) axis. When this happens and continues over time, we call it “allostatic load” to refer to the wear and tear on the body, resulting from the chronic overuse and imbalance of the “mediators” (McEwen, 2018). However, what really affects our health and well-being are the more subtle, gradual, and long-term influences from our social and physical environment, which contribute to allostatic load and overload through the same biological “mediators” that help us adapt and keep us alive, contributing also to shape our brains (McEwen, 2018). Therefore, the hormones have a fundamental role in the adaptation of the individual to the environment that can fluctuate in abiotic and biotic conditions over both short- and longer-term periods. Moreover, hormonally mediated behavioral variation among and behavioral flexibility within individuals is important for adjusting behavior to variation in environmental and social conditions (Ketterson et al., 2009; Hau and Goymann, 2015). With the new bioinformatics tools and the availability of many animal genomes, pieces of research have been discovering the way hormones influence the cellular transcriptome in order to define those genes and gene networks on which hormones act to modify cellular function and behavior (Schlinger, 2018). Since the first studies (Figure 2) on the role of hormones in behavioral endocrine, many works have been produced to provide evidence of the influence of sex steroid hormones, testosterone (T), estradiol (E2), and progesterone (P), as well as the hormones oxytocin (OXT) and vasopressin (AVP) on cortical and subcortical regions, implicated in emotional and cognitive processing (Callard et al., 1978; McEwen, 1981; Beltz and Moser, 2019; Kreta and De Gelder, 2021). In the traditional view, bodily glands under the control of various brain-pituitary factors secrete the hormones. The recent research on the social-sexual steroids hormones and neuropeptides has broadened this view of hormonal actions and the role that hormones play in the central nervous system (CNS), influencing neural circuit development, sex-specific behaviors, and sexual differentiation of mammalian brain from the fetal-neonatal period to puberty and adult life (Hines, 2011; McEwen and Milner, 2017). Moreover, the last scientific evidence (Torrens-Mas et al., 2020) shows that sex hormones may play an important role in the development and progression of several brain pathologies, including neurodegenerative diseases, such as Alzheimer's disease (AD) and Parkinson's disease (PD), stroke or brain cancer. T and E2 are both mainly synthesized in the testes and mammalian ovaries, and exert developmental and activational effects on the CNS (Bos et al., 2012; Toffoletto et al., 2014). Testosterone is also secreted, although in small quantities, by the ovaries in women and by the adrenal glands in both sexes. In premenopausal women, estrogens are produced primarily in the ovaries, corpus luteum, and placenta, although a small but significant amount of estrogens can also be produced by non-gonad organs, such as the liver, heart, skin, and brain (Cui et al., 2013). Progesterone is mainly secreted by the corpus luteum in the ovary during the second half of the menstrual cycle. Synthesis of OXT and AVP occurs mainly in the supraoptic nuclei (SON) and paraventricular nuclei (PVN) of the hypothalamus, where magnocellular neurons project to the posterior pituitary to release OXT and AVP in the bloodstream to exert their peripheral effects. Instead, parvocellular neurons in the PVN synthesize neuropeptides that project to diverse target regions within the CNS to exert synergistic behavioral and psychological effects, leading to the coordination of brain and body states (Landgraf and Neumann, 2004). In addition, a small portion of magnocellular neurons in the hypothalamus also projects to various regions in the CNS (Zhang and Hernandez, 2013). The hormones and neuropeptides, which were the prior regulators of sexual behavior in evolutionary distant species, have gained a more encompassing role through mammalian evolution, as the sustained importance of parental behavior toward offsprings increased. Hence, the simple sexual-regulatory actions of OXT, AVP, T, and E2 gradually extended to more complex social behavior (Bos et al., 2012), such as bonding between mothers and infants (Fries et al., 2005; Feldman et al., 2007; Neumann, 2009; Bos et al., 2010), partner bonding (Ditzen et al., 2009), social and emotion recognition (Bielsky and Young, 2004; Kirsch et al., 2005; Guastella et al., 2008; Unkelbach et al., 2008; Marsh et al., 2010; van Wingen et al., 2010; Veening and Olivier, 2013) and aggression between conspecifics (Archer, 2006; Mehta and Beer, 2009). Furthermore, considering also the particular attention that Hildegard of Bingen dedicated to women's health and evaluating the importance that the abbess dedicates to sex differences, we focused our interest mainly on ovarian hormones and their role in the modulation of social behavior and emotions, giving special emphasis to estrogens. We secondly focused also on the role of OXT and its receptor, whose effect and expression, in relation to behavior, is potentiated by estrogens (McCarthy, 1995; Acevedo-Rodriguez et al., 2015).

## Estrogen and Progesterone Receptors in the Regulation of Social Behaviors

The ovarian hormones, among the others, establish and maintain a specific neuroendocrine milieu through which the brain structure and function are modulated across a woman's life span. In fact, as shown in several works, both estrogen (E) (estrone, 17b-estradiol, and estriol) and progesterone (P) exert structural and functional trophic effects from early brain development throughout adolescence and adulthood by acting *via* classical nuclear receptors, as well as non-classical membrane-associated receptors. The sex steroid receptors (SSRs) are transcription factors mainly localized in the cytoplasm but also to the cell membrane, which regulate the expression of target genes by binding to specific sequences present at the level of DNA called “sex steroid response elements” (SSRE). These are nucleotide sequences specifically recognized by the hormone-receptor complex (Contrò et al., 2015). Interestingly, the well-characterized estrogen receptor (ER) and progesterone receptor (PR) are localized in brain regions, which are involved in emotional and cognitive regulation (Brinton et al., 2008; Wharton et al., 2012; Toffoletto et al., 2014). ER is expressed in two forms: ERα and ERβ. When the E binds the isoforms, they form homo and heterodimers that bind the DNA on the functional estrogen response elements (EREs) within the transcription regulatory region of specific genes, regulating the synthesis and metabolism of many neurotransmitters and neuropeptides, their receptors, and transporters. They also play a role in non-genomic cell signaling (the so-called rapidness of action) (Vargas et al., 2016; Dovey and Vasudevan, 2020). Therefore, ERs are essential for CNS development by regulating neuronal and glial proliferation, migration, differentiation, activation, and apoptosis of neurons (Talarowska et al., 2019). By the use of techniques, such as RT-PCR and Northern and Western blot analyses, it was found that many genes are estrogen regulated. However, among them, only a small number have been shown to possess EREs within the transcription regulatory region. In mammals, these genes include transcription factors, such as *JUN, FOS, PGR*, and *TP53*; intracellular signaling molecules, such as *HRAS, BCL2*, and *BRCA1*; enzymes, such as *CHAT, NQO1*, and *CKB*; secreted proteins, such as *LTF, SCGB1A1, OVGP1, C3*, and *AGT*; hormones, such as *LHB, OXT, PRL*, and *AVP*; membrane proteins, such as SNAT2 and VEGFA; the mitogen TFF1; and the protease CTSD. These genes are assumed to directly mediate various E actions in normal tissues, as well as in cancer and other diseases (Ikeda et al., 2015). Experiments of gene targeting in rats have shown that ERα contributes to the regulation of socio-sexual behaviors in both sexes (Le Moene et al., 2019). It is known that expression of the ERα gene is controlled by multiple promoters located upstream of the first-coding exon. In rats, the ERα gene transcript from the 0B promoter, which corresponds to the C promoter in humans and mice, is expressed in brain areas involved in sociosexual behaviors, such as the bed nucleus of the stria terminalis (BNST), the medial preoptic area (MPOA), and hypothalamic and amygdaloid nuclei, as well as in the anterior pituitary, ovary, and uterus. The ERα level is correlated with differences in the magnitude of expression of these behaviors between the sexes and among individuals within the same sex (Matsuda, 2014). In humans, ERα is higher in women in the diagonal band of Broca and in the medial mammillary nucleus, the suprachiasmatic nucleus, and the ventromedial nucleus. Conversely, ERα levels are higher in men in the sexually dimorphic nucleus of the MPOA, paraventricular nucleus, and lateral hypothalamic area. ERα is present in neurons, astrocytes, plexus choroideus, and other non-neuronal cells with some areas characterized by dimorphic distribution (Kruijver et al., 2002). The ERβ isoform is highly expressed in the claustrum, cerebral cortex, hippocampal formation, and in the central amygdala, which is responsible, to a certain extent, for the well-established anxiolytic effects of E and may modulate the level of arousal. ERβ phosphorylates and activates intracellular second messenger proteins and regulates protein expression of genes involved in neurological functions. It also promotes neurogenesis, modulates the neuroendocrine regulation of stress response, confers neuroprotection against ischemia and inflammation, and reduces anxiety- and depression-like behavior (Vargas et al., 2016). As demonstrated in animals, as well as in humans, both isoforms contribute to the estrogenic regulation of sexually dimorphic brain formation and sex-typical expression of social behaviors (Ogawa et al., 2020). Likewise, PRs are known to exist in two molecular forms, commonly designated as “A” and “B” forms. PRA and PRB are most abundant in the amygdala, cerebellum, cortex, hippocampus, and hypothalamus of rats (Toffoletto et al., 2014). In mammalian, the sexually dimorphic regulation of PR isoforms in the hypothalamus and preoptic area is functionally relevant for the control of sexual behavior and anxiety, as well as for the production of somatostatin and OXT receptors. In female brain, PRs mainly control reproductive behavior and are involved in the regulation of myelination and its repair after traumatic injury, neurogenesis and regeneration, inflammation, cognitive functions, and mood (Vegeto et al., 2020). In rat experiments, E selectively induces PRA in the cerebellum. Similarly, in the rat hippocampus and olfactory bulb, E2 induces PRA isoform expression, whereas P does not affect the expression of any PR isoforms (Brinton et al., 2008). The scientific evidence aforementioned shows the importance of ER and PR in controlling the molecular mechanism of the brain and underlying the regulation of the social behavior in relation to sex.

## The Origin of Disease in Hildegard of Bingen's Medicine: the Role of HPA Axis

### The Disease's Origin in Hildegard of Bingen: The Role of the Senses

In medieval medicine, the complexio or the proportion between the humors that make up the body varies from one person to another but also in the same individual, as a function of the human being environment or macrocosm-microcosm relationship, which Hildegard of Bingen explained in her writings (Calef, 1997; Crisciani and Pereira, 2003) with the image of the winds. Using this symbol, in fact, Hildegard of Bingen pushed us to consider the interaction between the two complex systems in a fluid and dynamic way, which includes the physiological part, but even more the sensory one (Hildegard of Bingen would speak of soul). Health corresponds to the balance between humors (flegmata and livores in Hildegard). The disease, on the other hand, arises from the misalignment between micro and macrocosm, and it is reflected in the humoral imbalance. In line with this view, the abbess describes the disease in *Causae et Curae* in the following way:

*When the soul of a man and a woman perceives something that can harm them, or their body, the heart, the liver, and the vessels contract. It is then that a sort of haze forms in the heart, darkening the heart to the point of making a human being sad. After sadness, anger rises. If a human being understands where sadness comes from, then this mist of sadness that rises from the heart produces a hot smoke in all humors, warming the bile, moving it, and so the bile acid makes a dull anger rise, contained (bitterness). If the human being does not let his or her anger erupt, the bile will calm down again. If, on the other hand, the anger does not calm down, then this gas will invade the black bile, exciting it and it will spread a black haze. This will go up to the brain, madness will take hold of it, and it will descend toward the intestine; here, it will destroy the blood vessels and bowels and will make the human being totally insane. The human being will forget and will let anger to explode, and, for this, he or she will fall into great diseases. Thus, the bile and the black bile can make human beings sick (Causae et Curae. Hildegard of Bingen)*.

In describing the origin of the disease, Hildegard of Bingen explained that it is the soul's job to recognize what we name the non-self or whatever is physiologically and psychologically harmful for the body. With attention to the soul, Hildegard of Bingen gave a broader meaning to the non-self by introducing the role of senses. In describing the contraction of vessels, heart, and liver, after the recognition of the non-self, the abbess refers to adaptation or stress syndrome and to the complex mechanism of emotions. Emotions are associated with feelings, which represent their internalization and are able to cause physiological changes in our internal environment. Hildegard of Bingen described this process as a fog that invades the heart and causes sadness. The heart, she said, is *the seat of the soul and is the door from which good and bad thoughts come out*. From feelings, therefore, we move on to thoughts and then to action and to behavior, as confirmed by actual affective neuroscience studies (Damasio, 2010).

### Hypothalamic-Pituitary-Adrenal Axis and Organism-Environment Interactions

Social-environmental stressors, which include events like social conflict, isolation, rejection, and exclusion, are represented in brain regions that play a role in processing experiences of social and physical threat, pain, and interoceptive. These brain regions, in turn, project to lower-level regions that upregulate systemic inflammatory activity *via* several non-mutually exclusive pathways. These include the hypothalamic-pituitary-adrenal (HPA) axis (Slavich and Sacher, 2019), which leads to the production of glucocorticoids (GC, cortisol in primates and corticosterone in rodents species), necessary to mediate the adaptation to changes in environmental (e.g., temperature, toxic food, infections) and internal (inflammation, tissue damage) conditions. HPA axis is a complex neuroendocrine system that functions synergistically with the locus coeruleus/norepinephrine autonomic nervous system in response to stress and in a circadian rhythm (Fragkaki et al., 2017). The secretion of corticotropin-releasing hormone (CRH) from the PVN of the hypothalamus due to changes in physical, biochemical, and/or physiological factors causes activation of anterior pituitary gland, which increases its release of adrenocorticotropic hormone (ACTH). ACTH, in turn, stimulates adrenals to produce GC, which travel through the bloodstream and cross the blood-brain barrier to activate glucocorticoid receptors (GRs) throughout the brain. Cortisol regulates metabolism, blood glucose levels, immune responses, anti-inflammatory actions, blood pressure, and emotion regulation (Petrescu et al., 2018; Sroykham and Wongsawat, 2019). While this release is critical to the enhancement of long-term memories associated with the event, glucocorticoid actions in the prefrontal cortex (PFC) impair working memory. Therefore, it is reasonable that dysregulation of the CRH may contribute to the pathophysiology of the major depressive disorders (MDD), as several works show (Aihara et al., 2007; Ishitobi et al., 2012; Menke, 2019) that that could be the scientific interpretation of the madness described by Hildegard of Bingen in her writings (Calef, 1997).

## The Estrogen's Role in the Regulation of HPA Axis

### Sex Difference in HPA Regulation

The body's response to environmental stimuli and, in general, the adaptation or stress response is different between men and women (Kudielka and Kirschbaum, 2005; Oyola and Handa, 2017; Heck and Handa, 2019). Experiments in rats suggest the E modulates, in the PFC, the factors that contribute to stress-induced impairments. The mechanisms by which E does this are not known, but several intriguing possibilities exist (Shansky and Lipps, 2013). Sex differences in the expression of CRH are reported to be higher in females than males in certain brain regions. Precisely, in some studies, it was found that CRH expression is higher in female than male rodents in the PVN and, because in this region initiates the HPA axis, increased CRH expression in the PVN of females may explain why levels of corticosterone are higher in female than male rodents (Bangasser and Wiersielis, 2018). Outside of the PVN, increased CRH expression has been reported by some to be elevated in the central nucleus of the amygdala (CeA) in female relative to male rats after electric foot shock, whereas psychological stress significantly increased CRH mRNA expression only in males (Iwasaki-Sekino et al., 2009). In another work, in the oval nucleus of the bed nucleus of the stria terminalis (BSTov), male rats showed an increase in CRH mRNA content, whereas, in the CeA, both sexes revealed similar increases in CRH mRNA (Sterrenburg et al., 2012). At the receptor level of CRH, there are sex differences in receptor expression, distribution, trafficking, and signaling, and many, but not all, of these sex differences have been linked to increased female CRH sensitivity (Bangasser et al., 2010). Steroid hormones, such as testosterone and its metabolites, control the HPA-axis gene (Crhr1, Crhr2, Nr3c1, Crh and Fkbp5) by regulating its gene transcription (Ludwig et al., 2019). E2 treatment in ovariectomized females of rhesus monkeys increased CRH expression in the PVN (Roy et al., 1999). Membrane estrogen receptors that initiate intracellular signaling cascades can also regulate CRH neurons. Both isoform ERα and ERβ regulate the human CRH gene through similar pathways (Chen et al., 2008; Weiser and Handa, 2009; Bangasser and Wiersielis, 2018) through E2 binding (Lalmansingh and Uht, 2008). A recent study on rats has suggested that HPA inhibition by long-term androgen treatment in supraphysiological doses leads to less resilience and higher susceptibility to depression-like symptoms. This appears to be associated with altered expression of HPA-axis genes in conjunction with CRH regulation by ER-β (Goldstein et al., 2005; Ludwig et al., 2019) as demonstrated also by Handa et al., which showed a modulatory effect on HPA axis by 3β-Diol (a testosterone's metabolite) (Handa et al., 2011), although the neurological mechanism underlying the ability of ERβ to alter HPA reactivity is not completely clarified. Moreover, recent findings have suggested that GR's sex-dependent regulation of PVN CRH may depend upon differences in the GR transcriptional machinery and an underlying influence of E2 levels in females (Heck et al., 2020). Because E levels are generally higher in women than in men, women show different stress response compared to men, which may be one of the reasons why women have pronounced rates of stress-related disorders, panic disorder, major depression, and fibromyalgia (FM) (Kudielka and Kirschbaum, 2005; Alves et al., 2016). However, mice models of depression show that overexpression of ERα in the nucleus accumbens (NAc) increases behavioral resilience in both sexes, but the downstream transcriptional mechanism of ERα action in males and females is different (Lorsch et al., 2018). This would explain why most people when exposed to stress do not develop depression. Sexual dimorphism has been implicated also in the risk, progression, and recovery from numerous neurological disorders. Accumulating evidence suggests that the neuroprotective effect of E, among its wide range of effects within CNS, could be one of the most important in the observed differences between men and women, with its neuroprotective effect being one of the most important (Czlonkowska et al., 2005).

### Is the HPA Response to Emotional Stress Mediated by ER?

Affective neuroscience recognizes the affective and conscious dimension of the homeodynamic regulation of the whole body. This background consciousness is mediated and integrated by brainstem diencephalic and limbic structures, such as thalamus, cingulum, insula, and involves the complex mechanism of emotions (Damasio, 2010). From the brain as we have described before, hormonal signals will define the changes at the metabolic, immune, and endocrinological levels, as it is evident in emotional trauma. In this regard, there are scientific works documenting the relationship between childhood trauma and chronic pain and emotional symptoms in adulthood, albeit physiological mechanisms mediating this link have not been elaborated (Tiwari and Gonzalez, 2018). For example, in one study of 179 adults with FM, it examined the mediating role of the cortisol profile in the linkage between childhood maltreatment and pain and emotional symptoms. Childhood neglect predicted a flattened cortisol profile, which, in turn, predicted elevated daily pain and emotional symptoms. The cortisol profile partially mediated the neglect-symptom relation. This would mean that early maltreatment might exert enduring effects on endocrine regulation that contributes to pain and emotional symptoms in adults with chronic pain (Yeung et al., 2016). In other words, it means that childhood trauma and the inability by the children to understand and manage it, therefore the inability to lower the cortisol level, can permanently compromise their ability to react to external stimuli up to the point of developing, in adulthood, syndromes such as FM. As we will describe later, this could be explained through the epigenetic modification of the ER gene during childhood that shows up in adulthood, under a stressful condition, such as cortisol imbalance. Because cortisol is important for regulating the immune system (Diaz-Jimenez et al., 2021), this chronic stress can result in impaired communication between the immune system and the HPA axis. The impaired communication is, in turn, linked to the development of numerous physical and mental health conditions, including chronic fatigue, metabolic disorders, depression, and immune disorders.

## The Relationship Between Microcosm and Macrocosm Explained Through the Epigenetic of Estrogen and Oxytocin

Hildegard of Bingen describes the interdependent relationship between organisms and environment through a circular dynamic, which is mediated by temperament, senses, and emotional behavior resulting from experience and personal history. The emotional experience conditioned by the innate temperament results in a change of internal milieu (Damasio, 2010), described by Hildegard as “raising livores,” with a consequent humoral imbalance. The sex-specific relationship between macrocosm and microcosm, expressed by Hildegard through temperaments, leads us to investigate how the environment is able to act on the individual physiological and psychological balance and how the proportion between male and female sex hormones can influence the origin and development of chronic diseases through epigenetic (Migliore and Nicoli, 2018). Starting from this observation, we evaluated the role of E and its receptors in the transduction of environmental changes into behavior. This aspect is important to explain the interaction and interdependence between microcosm and macrocosm and to understand how the emotional relationship can change the DNA. Therefore, in order to explain how the socio-environmental changes can modulate the brain and, consequently, the individual response to those changes and behavior, we reviewed the last scientific works, describing the role of the epigenetic in the modification of the E, OXT, and their receptors, given their role in the regulation of emotions. Furthermore, we also explored the capability of emotion to induce epigenetic modification of the ER and OXT receptor.

### Role of Epigenetic Modification of E and ER in the Social Behavior

Alteration of ERα gene activity, in particular brain areas, by epigenetic mechanism, such as histone modifications and methylation, appears to play central roles in the lasting regulation of ERα expression in response to the hormonal, social, and physiological environment during development, between the sexes and among individuals within the same sex (Francis et al., 1999; Matsuda, 2014). Moreover, data demonstrate the potential for E to influence gene methylation on the promoters of its own and related receptors in discrete brain regions of the developing brain (Nugent et al., 2011). Considering the role of ER in the social-sexual behavior and its role in the HPA axis regulation described before, it is obvious the importance of the epigenetic modification in the transduction of environmental changes into behavior. Still, emerging evidence shows that hormones can also alter epigenetic factors to regulate behavior. For example, experimental studies showed that E2 treatment during learning alters several epigenetic factors in the hippocampus, causing increased histone acetylation, reduced expression of the histone deacetylase HDAC2, and increased expression of DNA methyltransferase DNMT3B, culminating in enhanced memory. Hence, epigenetic factors can modify sensitivity to hormones by altering hormone receptor expression, and hormones can regulate epigenetic factors by recruiting epigenetic regulators to DNA (Baumbach and Zovkic, 2020). The bidirectional nature of this relationship suggests that the ability of hormones to regulate certain forms of behavior may depend on their ability to induce changes in the epigenome. In addition, animal experiments show that neonatal E2 establishes sex differences in the methylation levels of the ER-α, ER-β, and PR gene promoter regions, in neurons in the POA and mediobasal hypothalamus (MBH) and that these sex differences are maintained into adulthood, presumably altering expression of the gene and influencing adult brain hormonal sensitivity to regulate behavior (Schwarz et al., 2010). This, in our opinion, would confirm the Hildegard of Bingen's observation regarding the role playing by sex difference on the mechanism by which the environment acts on the human microcosm.

### The Role of Epigenetic Modification of OXT Gene and Receptor in the Social Behavior

Moreover, considering also the role of OXT in the modulation of empathy showed by several studies and the OXT estrogen's modulation (McCarthy, 1995), we were wondering if epigenetic modification of the OXT gene or OXT receptor gene could have any interesting effect on behavior. In a recent study, the relationship between the OXT gene methylation and empathy in mothers of children in early childhood has been examined. Researchers used the Interpersonal Reactivity Index to assess cognitive and affective dimensions of empathy of 57 mothers who participated in the study. Genetic data were collected *via* saliva samples and analyzed to quantify DNA methylation of the OXT gene. The results showed a positive correlation between OXT gene methylation and personal distress, an aspect of affective empathy (Hiraoka et al., 2021). This was also confirmed in another recent study, where it has been found that greater OXT DNA methylation, presumably linked to lower OXT expression, is associated with greater anxious attachment and a reduced ability to recognize emotional facial expressions. It was also found that, within the brain, greater OXT DNA methylation is associated with reduced neural activity within brain regions important for social cognitive functioning, and associated with reduced gray matter volume within the right fusiform gyrus, a brain region important for face processing and social cognition (Haasa et al., 2016). Other emerging studies also showed that the DNA methylation of OXT receptor (OXTRm) would play a role in the regulation of social relationship. In fact, it was showed that increased OXTRm impairs social, cognitive, and emotional functioning, while decreased OXTRm would lead to specific patterns of impairment related to mood and anxiety disorders (Maud et al., 2018). In another study using a developmental neuroimaging epigenetics approach in a large sample of infants (*N* = 98), it was examined whether OXTRm is associated with neural responses to emotional expressions. Critically, infants with higher OXTRm showed enhanced responses to anger and fear and attenuated responses to happiness in the right inferior frontal cortex, a region implicated in emotion processing through action-perception coupling (Krol et al., 2019). All of these data show, as for the epigenetic modulation of E and its receptor, the role of DNA modification of OXTR in the regulation of social behavior and relationship, confirming the importance of the epigenetic modification in the regulation of the behavior.

## Hildegard of Bingen's Insights: the Role of the Brain in the Environment-Organism Interaction

The complex mechanism of emotions and the feelings associated with them become brain maps that, over time, define our behaviors and personality traits (Damasio, 2010). Hildegard of Bingen describes this process *as a fog that surrounds the heart* and *which rises to the brain*, where it affects the nature of thoughts. This fog is actually attributable to the process mediated by our nervous system that connects the emotional experience to the modification of the mental processes underlying behavior. The circular relationship between microcosm and macrocosm starts from the environment and returns to it through the attitudes that human beings develop by emotional, intellectual, and social intelligence's combination. The brain maps that are gradually formed based on acquired experience, modify thoughts about the environment and ourselves. For Hildegard of Bingen, these thoughts lead to illness or, on the contrary, can allow us to maintain our state of health. This is how she related thoughts to the pathogenetic process in the book of Liber Divinorum Operum:

…*For these reasons, in many cases, after being so modified, (thoughts) enter the liver of a human being, where his or her science is evaluated coming from the brain and regulated by the forces of the soul[…] when the humors with their humidity flood the human being's chest beyond measure and moisten the liver, and, for this reason, innumerable different thoughts arise in the human being, and […] the humors going up to the brain, poison him or her, […] making him or her chronically ill (*Crisciani and Pereira, 2003*)*.

The brain, therefore, is an organ that transforms the mind's processes life, which is related to the organism-environment relationship, in structures. This relationship is different for each individual, since it is a synthesis of the dynamic relationships between brain, body, and environment, thus contributing to define personal subjectivity, which, in turn, also affects the pathogenetic process. Since the brain is, ultimately, a relational organ, embedded in the significant interactions of a living being with its environment, the interface, which we want to define, must necessarily interact with the brain itself, as E would demonstrate, given its action in areas of the brain concerned with the control of mood, mental state, cognition, emotion, and behavior (Haasa et al., 2016).

## Estrogen Receptor as a Potential Example of Biodynamic Interface?

### Epigenetic Modification of ER and OXT in Response to Social Stress

If we translate the above theories into a more molecular level, could we find bio-molecular mechanisms underlying those concepts? We described the importance of the E and ER in the modulation of the socio-sexual behavior. Above, we also reported some studies showing how alteration of ERα gene activity, in particular brain areas by epigenetic mechanism, plays central roles in the lasting regulation of ERα expression in response to the hormonal, social, and physiological environment during development between the sexes and among individuals within the same sex. Moreover, we also reported data showing the potential for E2 to influence gene methylation on the promoters of its own and related receptors in discrete brain regions of the developing brain. Accordingly, we focused our interest on recent works about the link between emotional stress and epigenetic modification of ER. In rodent adult offsprings of mothers that exhibited high licking and grooming activity, they had increased expression of ERα mRNA in the MPOA of the hypothalamus. This increased expression was associated with less methylation at the ERα 0/B promoter. Meanwhile, the ERα promoter in offsprings from low-licking and grooming mothers was hypermethylated (Wilson et al., 2008). Other animal studies confirmed changes in DNA methylation in ERα upon early-life adversity (Weaver et al., 2004). Nevertheless, in humans, there is only one study to suggest that childhood adversity might epigenetically modify the ERα shore in women. In this study, Fiacco and colleagues (Fiacco et al., 2019) proposed DNA methylation in the Erα shore as one possible mechanism linking childhood adversity and risk of psychopathology in women. Notably, they found a dose-dependent association between early-life adversity and the overall ERα shore methylation as well as for some single CpGs in a non-clinical sample of healthy adult women. Thus, they speculated that this effect could be even stronger in clinical populations and pathologies, such as depression, which is thought to be linked to both E actions and early-life adversity. However, further studies are needed to gain a better understanding of why some individuals remain healthy, and others develop psychopathologies in the face of childhood adversity. As observed for ER, data also show that DNA methylation of OXT is modified in response to early childhood experience and social stress (Unternaehrer et al., 2012; Essex et al., 2013).

### SIRT1's Role in the Epigenetic Regulation of Estrogen Receptor in the Behavioral Response to Pain Conditions

Trying to identify such biodynamic interfaces capable of mediating the transmission of messages between the environment and the body and *vice versa*, it will be of interest to determine the mechanisms that link the environmental cues to patterns of epigenetic modification in the ERα promoter and OXT. In this respect, it is worth considering the human protein SIRT1, a class III HDAC, which regulates ERα expression and responds to various environmental stressors and natural compounds. Inhibition of SIRT1 activity by sirtinol suppresses ERα expression through disruption of basal transcriptional complexes at the ERα promoter. This effect leads to inhibition of estrogen-responsive gene expression. *In vitro* experiments, mice have demonstrated that inhibition of SIRT1 deacetylase activity by either pharmacological inhibitors (sirtinol) or genetic depletion impairs ERα-mediated signaling pathways (Yao et al., 2010). Emotional disorders, depression, and high scores of sexual dysfunction are common comorbid conditions that further exacerbate the severity and chronicity of chronic pain, as observed in FM. However, individuals show considerable vulnerability to the development of chronic pain under similar pain conditions. In a study on male rat and mouse models of chronic neuropathic pain, it was identified SIRT1 in central amygdala is a key epigenetic regulator that controls the development of comorbid emotional disorders underlying the individual vulnerability to chronic pain (Zhou et al., 2020). In fact, researchers found that animals that were vulnerable to developing behaviors of anxiety and depression under the pain condition displayed reduced SIRT1 protein levels in their central amygdala, but not in those animals resistant to the emotional disorders. We could name the subjects resistant to the emotional disorders as the resilient. This study indicates that SIRT1 may serve as a potential therapeutic molecule for individualized treatment of chronic pain with vulnerable emotional disorders. Furthermore, the role played by SIRT1 as an epigenetic factor in ER in chronic pain and the effect described above confirms our hypothesis about the role of ER as a biodynamic interface capable of transducing the external stimuli into a behavioral effect.

## Discussion

Our study started with the aim of remarking the importance that psychosocial and environmental aspects have in the treatment of chronic disease, which can reach a better result going beyond the reductionist model and incorporating a more holistic view of the pathogenetic process. In an attempt to interpret the complexity of the organism-environment relationship, we referred to Arora's study where the concept of biodynamic interface is introduced. In fact, we thought that, through the properties of the interface and its modifications, it would be possible to evaluate the impact of the interaction between the environment and human physiology on the origin of disease and health, in agreement to Hildegard's vision (Figure 1) that inspired and guided our study.

In Hildegard of Bingen, each temperament is associated with a specific psychophysical balance, and defines the prevailing physical characteristics and the psychological, behavioral ones. The four temperaments, depending on the qualitative-quantity ratio between flegmata and livores (the four humors), respond differently to the solicitations coming from the inside and outside the organism. If the flegmata defines the innate temperament, livores, instead, are able to rise in response to stimuli to the point of involving the balance of the whole organism. This imbalance represents the disease. Considering the importance that Hildegard of Bingen attributed to the sex's difference and the role of livores, we hypothesized that her humoral theory suggests an intermediary role to sex hormones capable of defining both the organism-environment relationship and the evolution of the pathogenetic process. Therefore, our interpretation of humors could be found in modern neuroendocrinology (Figure 2), namely, in the hormones, and, specifically, in sex steroid hormones and neuropeptides, which have numerous behavioral, psychological, and organic effects on the brain and the body, in a sex different way, as well as in the development and progression of several brain pathologies, including neurodegenerative diseases, stroke or brain cancer. Moreover, in line with the humoral theory, today, studies also show that hormonally mediated behavioral variation among and behavioral flexibility within individuals is important for adjusting behavior to environmental stress.

Therefore, in light of the role of estrogen and progesterone in the CNS explained by modern scientific evidence, we would hypothesize that the magnitude of expression of the behavior, in relationship to the level of ER, as well as the sexually dimorphic regulation of PR isoform, may explain, through a neuroendocrinological perspective, part of the Hildegard of Bingen's original humoral theory, which hypothesizes the sex-specific regulation of social behavior.

Still, following the Hildegardian thoughts, we found a possible scientific interpretation of the madness resulting from the humoral imbalance, described from her as a fog, in the modern neuroscience, which demonstrates how the dysregulation of CRH in the HPA axis observed during chronic stress may contribute to the pathophysiology of major depressive disorders. In describing the stress syndrome, she emphasized the sex difference (different resilience) in the individual response to the external stimuli.

Scientific evidence shows today that, within CRH neurons, expression of CRH is reported to be higher in females than males in some brain regions. Furthermore, at the receptor level, there are sex differences in receptor expression, distribution, trafficking and signaling, and many, but not all, of these sex differences have been linked to increased female CRH sensitivity. Steroid hormones, such as testosterone and its metabolites, control the HPA-axis gene by regulating its gene transcription. Several works show also the involvement of ERs in this regulation of CRH expression by sexual hormones, although the neurological mechanism underlying the ability of ER to alter HPA reactivity is not completely clarified. In the HPA-axis regulation, hormonal signals modulate the changes at the metabolic, immune, and endocrinological level, but, as shown by clinical studies, the enduring cortisol imbalance for the inability to lower its level (a condition called distress) may permanently compromise the ability to react to external stimuli up to the point of developing syndromes such as FM.

The complexity of the organism-environment relationship in the Hildegardian vision, well-represented by the symbol of the wheel (Figure 3), also considers the changes that the environment is able to generate on male and female organisms and on their personality. We found a scientific interpretation of this in the role played by epigenetic in the modification of E, OXT, and their receptor, given their role also in the regulation of emotions. Therefore, considering the role of ER in the social-sexual behavior and in the HPA axis regulation and considering the role of OXT in the modulation of empathy, it is obvious the importance of the epigenetic modification in the transduction of environmental changes into behavior. Data show also that epigenetic factors can modify sensitivity to hormones by altering hormone receptor expression, and hormones can modulate epigenetic factors to regulate behavior by recruiting epigenetic regulators to DNA. Therefore, the bidirectional nature of this relationship suggests that the ability of hormones to regulate certain forms of behavior may depend on their ability to induce changes in the epigenome.

In perfect agreement with the latest research evidence in psychology and neuroscience, also, Hildegard of Bingen conceived the brain as an organ that integrates processes that occur between the organism and the environment. The brain is, ultimately, a relational organ embedded in the interactions of a living being with its environment. Therefore, the interface we want to define must necessarily interact with the brain itself as E would demonstrate, given its action in areas of the brain concerned with the control of mood, mental state, cognition, emotion, and behavior, besides its role in the HPA-axis regulation.

Furthermore, we explored the capability of emotion to induce epigenetic modification of the ER and OXT receptor, finding data that show the link between emotional stress and epigenetic modification of ER and OXT in the risk of developing psychopathology. In conclusion, in order to explain the idea of biodynamic interface capable to mediate the transmission of messages between the environment and the body and *vice versa*, we tried to understand how ER and OXT might dynamically operate with the environment in order to transduce the external signal stimuli into an organic and behavioral response. We found that human protein SIRT1, responding to various environmental stressors and natural compounds, regulates ERα expression. Moreover, SIRT1 was identified in central amygdala as a key epigenetic regulator that controls the development of comorbid emotional disorders underlying the individual vulnerability to chronic pain. This would indicate that SIRT1 might serve as a potential therapeutic molecule for individualized treatment of chronic pain with vulnerable emotional disorders. In conclusion, the role played by SIRT1 as an epigenetic factor in ER under stress conditions could confirm our hypothesis about the role of ER as a biodynamic interface capable of transducing the external stimuli into a behavioral effect.

## Conclusion

**Figure 5:**
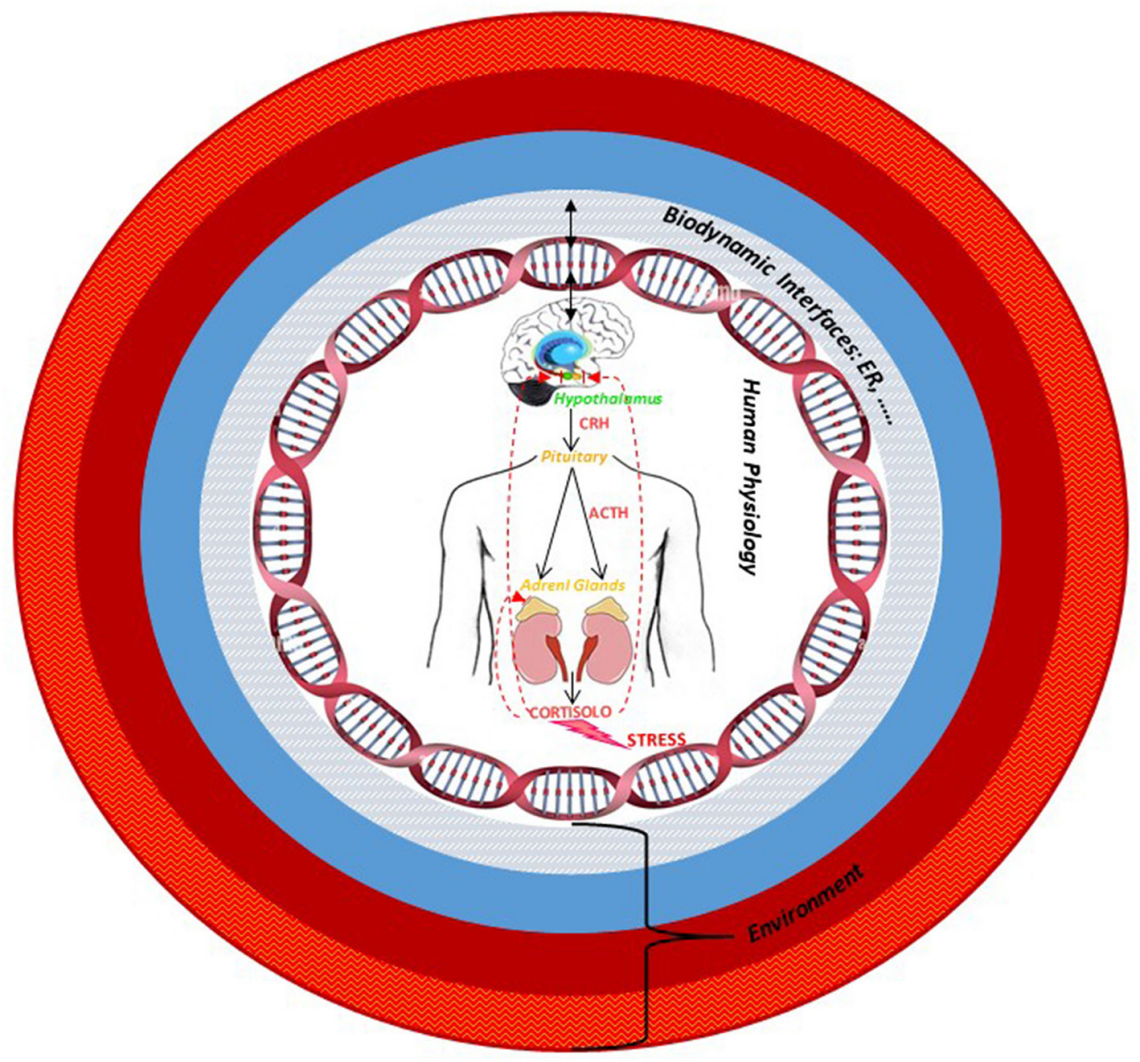
The multiple levels organization of human being–environment system: The estrogen receptor, as an example of biodynamic interface, modulates the interaction among external socio-environmental stressors, the body, and the consequent behavioral changes. The arrows indicate the bidirectional and dynamic interaction between the organism and the environment *via* the interface.

Through this review, we wanted to enhance Hildegard's insights and her anticipations with respect to current scientific evidence, giving importance in the pathogenetic process to the role of hormones, to the specific sex-gender difference, and to the authentic relationship that each individual establishes with the environment. Given the complexity described, in order to understand the disease, it is essential to read it, starting from the awareness that the body is a dynamic space where the organic, psychophysical, cultural, and spiritual dimensions merge. In this fluidity, each interface is also necessarily dynamic, since it can only reflect the multiplicity of interrelationships that include the integration of multiple levels of organization. Therefore, we hypothesized that identifying those interfaces from a molecular perspective could be fundamental in order to understand how to create a more complete and efficient model of approach to treatment and a model of disease prevention that is able to read the human-environment complexity. Under the light of Hildegard of Bingen's medicine, through the review of the last scientific works in the field of neuroendocrinology, which confirms part of her intuitions, we arrived to speculate that estrogen receptor, within the HPA axis' stress response, could be an example of a molecular interface, a factor suitable for reading and measuring the determinants of disease (included social, psychological, and environmental ones) (Figure 5). Considering its ability to modulate the interaction between the external socio-environmental stressors, the body, and the consequent behavioral changes, it could be a challenging factor to be taken into consideration during the disease diagnosis stress related for the interpretation of its molecular mechanism and, lastly, in the choice of a medical treatment. In the last decade, accumulating scientific evidence has suggested the role of estrogen in the neuronal plasticity and in neurodegeneration. Consequently, a full understanding of the molecular mechanism triggered by estrogen may provide profound and exciting possibilities for the future treatment of, for example, age-dependent diseases associated with the regulation of sexual hormone levels. We thought that besides ER, most likely, other factors may exist that could play as biodynamic interfaces and thus, we would like to encourage others to broaden their studies and interpretation to look for them.

## Data Availability Statement

The original contributions presented in the study are included in the article/supplementary material, further inquiries can be directed to the corresponding author/s.

## Author Contributions

SM and EM performed the literature searches and wrote and edited the manuscript. All authors contributed to the article and approved the submitted version.

## Funding

This work was supported by Mildes Group srl, an innovative startup born after 10 years of study, research, and experience with Hildegard of Bingen's natural remedies (www.thesauranaturae.com).

## Conflict of Interest

The authors declare that the research was conducted in the absence of any commercial or financial relationships that could be construed as a potential conflict of interest.

## Publisher's Note

All claims expressed in this article are solely those of the authors and do not necessarily represent those of their affiliated organizations, or those of the publisher, the editors and the reviewers. Any product that may be evaluated in this article, or claim that may be made by its manufacturer, is not guaranteed or endorsed by the publisher.

## References

[B1] Acevedo-Rodriguez A. Mani S. K. Handa R. J. (2015). Oxytocin and estrogen receptor β in the brain: an overview. Front. Endocrinol. 6, 160. 10.3389/fendo.2015.00160 26528239 PMC4606117

[B2] Aihara M. Ida I. Yuuki N. Oshima A. Kumano H. Takahashi K.. (2007). HPA axis dysfunction in unmedicated major depressive disorder and its normalization by pharmacotherapy correlates with alteration of neural activity in prefrontal cortex and limbic/paralimbic regions. Psychiatry Res. 155, 245–256. 10.1016/j.pscychresns.2006.11.002 17587554

[B3] Alves B. Zakka T. M. Teixeira M. J. Kaziyama H. H. Siqueira J. T. T. Siqueira S. R. D. T. (2016). Depression, sexuality and fibromyalgia syndrome: clinical findings and correlation to hematological parameters. Arq. Neuropsiquiatr. 74, 863–868. 10.1590/0004-282x20160141 27901249

[B4] Archer J. (2006). Testosterone and human aggression: an evaluation of the challenge hypothesis. Neurosci. Biobehav. Rev. 30, 319–345. 10.1016/j.neubiorev.2004.12.007 16483890

[B5] Arora M. Giuliani A. Curtin P. (2020). Biodynamic interface are essential for human-environment. Bioessays 42, e2000017. 10.1002/bies.202000017 32851694

[B6] Bangasser D. A. Curtis A. Reyes B. A. S. Bethea T. T. Parastatidis I. Ischiropoulos H.. (2010). Sex differences in corticotropin-releasing factor receptor signaling and trafficking: potential role in female vulnerability to stress-related psychopathology. Mol Psychiatry 877, 896–904. 10.1038/mp.2010.66 20548297 PMC2935505

[B7] Bangasser D. A. Wiersielis K. R. (2018). Sex differences in stress responses: a critical role for corticotropin-releasing factor. Hormones 17, 5–13. 10.1007/s42000-018-0002-z 29858858

[B8] Baumbach J. L. Zovkic I. B. (2020). Hormone-epigenome interactions in behavioural regulation. Horm. Behav. 118, 104680. 10.1016/j.yhbeh.2020.104680 31927018

[B9] Beltz A. M. Moser J. S. (2019). Ovarian hormones: a long overlooked but critical contributor to cognitive brain structures and function. Ann. N.Y. Acad. Sci. 1464, 1561180. 10.1111/nyas.14255 31639230

[B10] Bielsky I. F. Young L. J. (2004). Oxytocin, vasopressin, and social recognition in mammals. Peptides 25, 1565–1574. 10.1016/j.peptides.2004.05.019 15374658

[B11] Bizzarri M. (2020). “Revisiting the concept of human disease”, in Approaching Complex Diseases, Network-Based Pharmacology and Systems Approach in Bio-Medicine (Cham: Springer), 1–26. 10.1007/978-3-030-32857-3_1

[B12] Bos P. A. Hermans E. J. Montoya E. R. van Honk R. J. (2010). Testosterone administration modulates neural responses to crying infants in young females. Psychoneuroendocrinology 35, 114–121. 10.1016/j.psyneuen.2009.09.013 19819079

[B13] Bos P. A. Panksepp J. Bluthé R. M. van Honk J. (2012). Acute effects of steroid hormones and neuropeptides on human social–emotional behavior: a review of single administration studies. Front. Neuroendocrinol. 33, 17–35. 10.1016/j.yfrne.2011.01.002 21256859

[B14] Brinton R. D. Thompson R. F. Foy M. R. Baudry M. Wang J. Finch C. E.. (2008). Progesterone receptors: form and function in brain. Front. Neuroendocrinol. 29, 313–339. 10.1016/j.yfrne.2008.02.001 18374402 PMC2398769

[B15] Calef P. (1997). Cause et Cure delle Infermità. Palermo: Sellerio Ed.

[B16] Callard G. V. Petro Z. Ryan K. J. (1978). Phylogenetic distribution of aromatase and other androgen-converting enzymes in the central nervous system. Endocrinology 103, 2283–2290. 10.1210/endo-103-6-2283 748049

[B17] Calver A. D. Falmer A. A. Murray M. Strauss O. J. Streicher E. M. Thelma M. H.. (2010). Emergence of increased resistance and extensively drug-resistant tuberculosis despite treatment adherence, South Africa. Emerg. Infect. Dis. 16, 264–271. 10.3201/eid1602.090968 20113557 PMC2958014

[B18] Chen X. N. Zhu H. Meng Q. Y. Zhou J. N. (2008). Estrogen receptor-Alpha and -Beta regulate the human corticotropin-releasing hormone gene through similar pathways. Brain Res. 1223, 1–10. 10.1016/j.brainres.2008.05.043 18597742

[B19] Christakis N. A. Fowler J. H. (2007). The spread of obesity in a large social network over 32 years. N. Engl. J. Med. 357, 370–379. 10.1056/NEJMsa066082 17652652

[B20] Cockerham W. C. Hamburgy B. W. Oates G. R. (2017). The social determinants of chronic disease. J. Prev. Med. 52, S5–S12. 10.1016/j.amepre.2016.09.010 27989293 PMC5328595

[B21] Contrò V. Basile J. R. Proia P. (2015). Sex steroid hormone receptors, their ligands, and nuclear and non-nuclear pathways. AIMS Mol. Sci. 2, 294–310. 10.3934/molsci.2015.3.29425070950

[B22] Crisciani M. Pereira M. (2003). Libro delle Opere Divine. Milano: Mondadori Editore SPA.

[B23] Cui J. Shen Y. Li R. (2013). Estrogen synthesis and signaling pathways during ageing: from periphery to brain. Trends Mol. Med. 19, 197–209. 10.1016/j.molmed.2012.12.007 23348042 PMC3595330

[B24] Czlonkowska A. Ciesielska A. Gromadzka G. Kurkowska-Jastrzebska I. (2005). Estrogen and cytokines production - the possible cause of gender differences in neurological diseases. Curr. Pharm. Des. 11, 1017–1030. 10.2174/1381612053381693 15777251

[B25] Damasio A. (2010). Self Comes to Mind. Constructing the Conscious Brain. Milano: Ed Adelphi SPA.

[B26] Diaz-Jimenez D. Kolb J. P. Cidlowski J. A. (2021). Glucocorticoids as regulators of macrophage-mediated tissue homeostasis. Front. Immunol. 12, 669891. 10.3389/fimmu.2021.669891 34079551 PMC8165320

[B27] Ditzen B. Schaer M. Gabriel B. Bodenmann G. Ehlert U. Heinrichs M. (2009). Intranasal oxytocin increases positive communication and reduces cortisol levels during couple conflict. Biol. Psychiatry. 65, 728–731. 10.1016/j.biopsych.2008.10.011 19027101

[B28] Dovey J. L. Vasudevan N. (2020). Does GPER1 play a role in sexual dimorphism?. Front. Endocrinol. 11, 595895. 10.3389/fendo.2020.595895 33193108 PMC7661790

[B29] Essex M. J. Boyce W. T. Hertzman C. Lam L. L. Armstrong J. M. Neumann S. M.. (2013). Epigenetic Vestiges of early developmental adversity: childhood stress exposure and DNA methylation in adolescence. Child Dev. 84, 58–75. 10.1111/j.1467-8624.2011.01641.x 21883162 PMC3235257

[B30] Feldman R. Weller A. Zagoory-Sharon O. Levine A. (2007). Evidence for a neuroendocrinological foundation of human affiliation: plasma oxytocin levels across pregnancy and the postpartum period predict mother–infant bonding. Psychol. Sci. 18, 965–970. 10.1111/j.1467-9280.2007.02010.x 17958710

[B31] Fiacco S. Gardini E. S. Mernone L. Schick L. Ehlert U. (2019). DNA Methylation in healthy older adults with an history of childhood adversityfindings from the women 40+ Healthy Aging Study. Front. Psychiatry 10, 777. 10.3389/fpsyt.2019.00777 31708823 PMC6819958

[B32] Flanagan S. (1996). “Hildegard and the humors,” in Medieval Theories of Illness and Personality. Madness, Melancholy (The Limits of the Self, Graven Images), 14–23.

[B33] Fragkaki I. Cima M. Granic I. (2017). The role of trauma in the hormonal interplay of cortisol, testosterone, and oxytocin in adolescent aggression. Psychoneuroendocrinology 88, 24–37. 10.1016/j.psyneuen.2017.11.005 29156403

[B34] Francis D. Diorio J. Liu D. Meaney M. (1999). Nongenomic transmission across generations of maternal behavior and stress responses in the rat. Science 286, 1155–1158. 10.1126/science.286.5442.1155 10550053

[B35] Fries A. B. Ziegler T. E. Kurian J. R. Jacoris S. Pollak S. D. (2005). Early experience in humans is associated with changes in neuropeptides critical for regulating social behavior. Proc. Natl. Acad. Sci. U.S.A. 102, 17237–17240. 10.1073/pnas.0504767102 16303870 PMC1287978

[B36] Goldstein I. Meston C. M. Davis S. Traish A. (2005). Women's Sexual Function and Dysfunction: Study, Diagnosis and Treatment. London: CRC Press. 10.1201/b14618

[B37] Greene J. A. Loscalzo J. (2017). Putting the patient back together - social medicine, network medicine, and the limits of reductionism. N. Engl. J. Med. 377, 2493–2499. 10.1056/NEJMms1706744 29262277

[B38] Guastella A. J. Mitchell P. B. Dadds M. R. (2008). Oxytocin increases gaze to the eye region of human faces. Biol. Psychiatry 63, 3–5. 10.1016/j.biopsych.2007.06.026 17888410

[B39] Haasa B. W. Filkowskia M. M. Cochrana R. N. Denisonc L. Ishakd A. Nishitanic S.. (2016). Epigenetic modification of OXT and human sociability. Proc. Natl. Acad. Sci. U.S.A. 113, E3816–E3823. 10.1073/pnas.1602809113 27325757 PMC4941462

[B40] Handa R. J. Sharma D. Uht R. (2011). Role for the androgen metabolite, 5alpha androstane 3beta, 17beta Diol (3β-Diol) in the regulation of the hypothalamo-pituitary–adrenal axis. Front. Endocrinol. 2, 65. 10.3389/fendo.2011.00065 22649380 PMC3355903

[B41] Hau M. Goymann W. (2015). Endocrine mechanisms, behavioral phenotypes and plasticity: known relationships and open questions. Front. Zool. 12 (Suppl. 1), S7. 10.1186/1742-9994-12-S1-S7 26816524 PMC4722346

[B42] Heck A. L. Handa R. J. (2019). Sex differences in the hypothalamic-pituitary-adrenal axis response to stress: an important role for gonadal hormones. Neuropsychopharmacology 44, 45–58. 10.1038/s41386-018-0167-9 30111811 PMC6235871

[B43] Heck A. L. Thompson M. K. Uht R. M. Handa R. H. (2020). Sex-dependent mechanisms of glucocorticoid regulation of the mouse hypothalamic corticotropin-releasing hormone gene. Endocrinology 161, bqz012. 10.1210/endocr/bqz012 31754709 PMC7188085

[B44] Hines M. (2011). Prenatal endocrine influences on sexual orientation and on sexually differentiated childhood behavior. Front. Neuroendocrinol. 32, 170–182. 10.1016/j.yfrne.2011.02.006 21333673 PMC3296090

[B45] Hiraoka D. Nishitani S. Shimada K. Kasaba R. Fujisawa T. X. Tomoda A. (2021). Epigenetic modification of the oxytocin gene is associated with gray matter volume and trait empathy in mothers. Psychoneuroendocrinology 123, 105026. 10.1016/j.psyneuen.2020.105026 33130408

[B46] Ikeda K. Horie-Inoue K. Inoue S. (2015). Identification of estrogen-responsive genes based on the DNA binding properties of estrogen receptors using high-throughput sequencing technology. Acta. Pharmacol. Sin. 36, 24–31. 10.1038/aps.2014.123 25500870 PMC4571320

[B47] Ishitobi Y. Nakayama S. K. Kanehisa M. Higuma H. Maruyama Y. Ninomiya T.. (2012). Association of CRHR1 and CRHR2 with major depressive disorder and panic disorder in a Japanese population. J. Med. Genet. B Neuropsychiatr. Genet. 159B, 429–436. 10.1002/ajmg.b.32046 22467522

[B48] Iwasaki-Sekino A. Mano-Otagiri A. Ohata H. Yamauchi N. Shibasaki T. (2009). Gender differences in corticotropin and corticosterone secretion and corticotropin-releasing factor mRNA expression in the paraventricular nucleus of the hypothalamus and the central nucleus of the amygdala in response to footshock stress or psychological stress in rats. Psychoneuroendocrinology 34, 226–237. 10.1016/j.psyneuen.2008.09.003 18849120

[B49] Ketterson E. D. Atwell J. W. McGlothlin J. W. (2009). Phenotypic Integration and independence: hormones, performance, and response to environmental change. Integ. Comp. Biol. 49, 365–379. 10.1093/icb/icp057 21665827 PMC4012227

[B50] Kirsch P. Esslinger C. Chen Q. Mier D. Lis S. Siddhanti S.. (2005). Oxytocin modulates neural circuitry for social cognition and fear in humans. J. Neurosci. 25, 11489–11493. 10.1523/JNEUROSCI.3984-05.2005 16339042 PMC6725903

[B51] Kreta M. E. De Gelder B. (2021). A review on sex differences in processing emotional signals. Neuropsychologia 50, 1211–1221. 10.1016/j.neuropsychologia.2011.12.022 22245006

[B52] Krol K. M. Puglia M. H. Morris J. P. Connelly J. J. Grossmann T. (2019). Epigenetic modification of the oxytocin receptor gene is associated with emotion processing in the infant brain. Dev. Cogn. Neurosci. 37, 100648. 10.1016/j.dcn.2019.100648 31125951 PMC6969294

[B53] Kruijver F. P. Balesar R. Espila A. M. Unmehopa U. A. Swaab D. F. (2002). Estrogen receptor-alpha distribution in the human hypothalamus in relation to sex and endocrine status. J. Comp. Neurol. 454, 115–139. 10.1002/cne.10416 12412138

[B54] Kudielka B. M. Kirschbaum C. (2005). Sex differences in HPA axis responses to stress: a review. Biol. Psychol. 69, 113–132 10.1016/j.biopsycho.2004.11.009 15740829

[B55] Lalmansingh A. S. Uht R. M. (2008). Estradiol regulates corticotropin-releasing hormone gene (crh) expression in a rapid and phasic manner that parallels estrogen receptor-alpha and -beta recruitment to a 3',5'-cyclic adenosine 5'-monophosphate regulatory region of the proximal crh promoter. Endocrinology 149, 346–357. 10.1210/en.2007-0372 17947358 PMC2194609

[B56] Landgraf R. Neumann I. D. (2004). Vasopressin and oxytocin release within the brain: a dynamic concept of multiple and variable modes of neuropeptide communication. Front. Neuroendocrinol. 25, 150–176. 10.1016/j.yfrne.2004.05.001 15589267

[B57] Le Moene O. Stavarache M. Ogawa S. Musatov S. Ågmo A. (2019). Estrogen receptors αand βin the central amygdala and the ventromedial nucleus of the hypothalamus: sociosexual behaviors, fear and arousal in female rats during emotionally challenging events. Behav. Brain Res. 367; 128–142. 10.1016/j.bbr.2019.03.045 30928462

[B58] Lorsch Z. S. Eddie Loh Y. H. Purushothaman I. Walker D. M. Parise E. M. Salery M.. (2018). Estrogen receptor α drives pro-resilient transcription in mouse models of depression. Nat. Commun. 9, 1116. 10.1038/s41467-018-03567-4 29549264 PMC5856766

[B59] Ludwig B. Roy B. Dwivedi Y. (2019). Role of HPA and the HPG axis interaction in testosterone-mediated learned helpless behavior. Mol. Neurobiol. 56, 394–405. 10.1007/s12035-018-1085-x 29704202 PMC6204317

[B60] Marron B. M. (2014). A Space of Convergence: Hildegard of Bingen's Multivalent Understanding of the Body. Magistra, Academia.edu.

[B61] Marsh A. A. Yu H. H. Pine D. S. Blair R. J. (2010). Oxytocin improves specific recognition of positive facial expressions. Psychopharmacology 209, 225–232. 10.1007/s00213-010-1780-4 20186397

[B62] Matsuda K. I. (2014). Epigenetic changes in the estrogen receptor α gene promoter: implications in socio-sexual behaviors. Front. Neurosci. 8, 344. 10.3389/fnins.2014.00344 25389384 PMC4211403

[B63] Maud C. Ryan J. McIntosh J. E. Olsson C. A. (2018). The Role of Oxytocin Receptor Gene (OXTR) DNA Methylation (DNAm) in human social and emotional functioning: a systematic narrative review. BMC Psychiatry 18, 154. 10.1186/s12888-018-1740-9 29843655 PMC5975530

[B64] McCarthy M. M. (1995). Estrogen modulation of oxytocin and its relation to behavior. Adv. Exp. Med. Biol. 395, 235–245. 8713972

[B65] McEwen B. S. (1981). Neural gonadal steroid actions. Science 211, 1303–1311. 10.1126/science.6259728 6259728

[B66] McEwen B. S. (2018). Redefining neuroendocrinology: epigenetics of brain-body communicationover the life course. Front. Neuroendocrinol. 49, 8–30. 10.1016/j.yfrne.2017.11.001 29132949

[B67] McEwen B. S. Milner T. A. (2017). Understanding the broad influence of sex hormones and sex differences in the brain. J. Neurosci. Res. 95, 24–39. 10.1002/jnr.23809 27870427 PMC5120618

[B68] Mehta P. H. Beer J. (2009). Neural mechanisms of the testosterone-aggression relation: the role of orbito-frontal cortex. J. Cogn. Neurosci. 22, 2357–2368. 10.1162/jocn.2009.21389 19925198

[B69] Menke A. (2019). Is the HPA axis as target for depression outdated, or is there a new hope? Front Psychiatry. 10, 101. 10.3389/fpsyt.2019.00101 30890970 PMC6413696

[B70] Migliore L. Nicoli V. (2018). Epigenetics and gender- specific medicine. Ital. J. Gender-Specific Med. 4, 3–12. 10.1723/2968.29764

[B71] Moulinier L. (2003). Introduction, Beatae Hildegardis Cause et Cure. Berlin: Akademie Verlag. CXVII + 384p. 10.1524/9783050056098

[B72] Neumann I. D. (2009). The advantage of social living: brain neuropeptides mediate the beneficial consequences of sex and motherhood. Front. Neuroendocrinol. 30, 483–496. 10.1016/j.yfrne.2009.04.012 19416734

[B73] Nugent B. M. Schwarz J. M. McCarthy M. M. (2011). Hormonally-mediated epigenetic changes to steroid receptors in the developing brain: implications for sexual differentiation. Horm. Behav. 59, 338–344. 10.1016/j.yhbeh.2010.08.009 20800064 PMC3011040

[B74] Ogawa S. Tsukaharab S. Cholerisc E. Vasudevand N. (2020). Estrogenic regulation of social behavior and sexually dimorphic brain formation. Neurosci. Biobehav. Rev. 110, 46–59. 10.1016/j.neubiorev.2018.10.012 30392880

[B75] Oyola M. G. Handa R. J. (2017). Hypotalamic-pituitary-adrenal and hypothalamic-pituitary-gonadal axes: sex differences in regulation of stress responsivity. Stress 20, 476–494. 10.1080/10253890.2017.1369523 28859530 PMC5815295

[B76] Petrescu A. D. Kain J. Liere V. Heavener T. De Morrow S. (2018). Hypothalamus-pituitary-adrenal dysfunction in cholestatic liver disease. Front. Endocrinol. 9, 660. 10.3389/fendo.2018.00660 30483216 PMC6240761

[B77] Puchalski C. M. Vitillo R. Hull S. K. Reller N. (2014). Improving the spiritual dimension of whole person care: reaching national and international consensus. J. Palliat. Med. 17, 642–656. 10.1089/jpm.2014.9427 24842136 PMC4038982

[B78] Ross C. A. Margolis R. L. (2018). Research domain criteria: cutting edge neuroscience or galen's humors revisited? Mol. Neuropsychiatry 4, 158–163. 10.1159/000493685 30643789 PMC6323392

[B79] Roy B. N. Reid R. L. Van Vugt D. A. (1999). The effects of estrogen and progesterone on corticotropin-releasing hormone and arginine vasopressin messenger ribonucleic acid levels in the paraventricular nucleus and supraoptic nucleus of the rhesus monkey. Endocrinology 140, 2191–2198. 10.1210/endo.140.5.6684 10218971

[B80] Schlinger B. A. (2018). Hormonal control of behavior: novel mechanisms and model organisms. J. Comp. Physiol. A Neuroethol. Sens. Neural Behav. Physiol. 204, 1–3. 10.1007/s00359-017-1226-0 29101455

[B81] Schwarz J. M. Nugent B. M. McCarthy M. M. (2010). Developmental and hormone-induced epigenetic changes to estrogen and progesterone receptor genes in brain are dynamic across the life span. Endocrinology 151, 4871–4881. 10.1210/en.2010-0142 20702577 PMC2946142

[B82] Sears M. E. Genuis J. S. (2012). Environmental determinants of chronic disease and medical approaches: recognition, avoidance, supportive therapy, and detoxification. J. Environ. Public Health 2012, 356798. 10.1155/2012/356798 22315626 PMC3270432

[B83] Shansky R. M. Lipps J. (2013). Stress-induced cognitive dysfunction: hormone-neurotransmitter interactions in the prefrontal cortex. Front. Hum. Neurosci. 7, 123. 10.3389/fnhum.2013.00123 23576971 PMC3617365

[B84] Slavich G. M. Sacher J. (2019). Stress, sex hormones, inflammation, and major depressive disorder: extending social signal transduction theory of depression to account for sex differences in mood disorders. Psychopharmacology 236, 3063–3079. 10.1007/s00213-019-05326-9 31359117 PMC6821593

[B85] Sroykham W. Wongsawat Y. (2019). Effects of brain activity, morning salivary cortisol, and emotion regulation on cognitive impairment in elderly people. Medicine 98, e16114. 10.1097/MD.0000000000016114 31261527 PMC6616250

[B86] Sterrenburg L. Gaszner B. Boerrigter J. Santbergen L. Bramini M.. (2012). Sex-dependent and differential responses to acute restraint stress of corticotropinreleasing factor-producing neurons in the rat paraventricular nucleus, central amygdala, and bed nucleus of the stria terminalis. J. Neurosci. Res. 90, 179–192. 10.1002/jnr.22737 21922520

[B87] Sweet V. (2006). “A fluid concept,” in Rooted in the Earth, Rooted in the Sky. Hildegard of Bingen and Premodern Medicine (New York, NY: Routledge Taylor & Francis Group), 93–123.

[B88] Talarowska M. E. Szemraj J. Kuan-Pin S. (2019). Expression of ESR1 and ESR2 oestrogen receptor encoding gene and personality traits-preliminary study. Prz Menopauzalny 18, 133–140. 10.5114/pm.2019.90804 31975979 PMC6970415

[B89] Tiwari A. Gonzalez A. (2018). Biological alterations affecting risk of adult psychopathology following childhood trauma: a review of sex differences. Clin. Psychol. Rev. 66, 69–79. 10.1016/j.cpr.2018.01.006 29433843

[B90] Toffoletto S. Lanzenbergerb R. Gingnellc M. Sundström-Poromaad I. Comascoa E. (2014). Emotional and cognitive functional imaging of estrogen and progesterone effects in the female human brain: a systematic review. Psychoneuroendocrinology 50, 28–52. 10.1016/j.psyneuen.2014.07.025 25222701

[B91] Torrens-Mas M. Pons D. G. Sastre-Serra J. Oliver J. Roca P. (2020). Sexual hormones regulate the redox status and mitochondrial function in the brain. pathological implications. Redox Biol. 31, 101505. 10.1016/j.redox.2020.101505 32201220 PMC7212485

[B92] Unkelbach C. Guastella A. J. Forgas J. P. (2008). Oxytocin selectively facilitates recognition of positive sex and relationship words. Psychol. Sci. 19, 1092–1094. 10.1111/j.1467-9280.2008.02206.x 19076479

[B93] Unternaehrer E. Luers P. Mill J. Dempster E. Meyer A. H. Staehli S.. (2012). Dynamic changes in DNA Methylation of stress-associated genes (OXTR, BDNF) after acute psychosocial stress. Transl. Psychiatry. 2, e150. 10.1038/tp.2012.77 22892716 PMC3432191

[B94] van Wingen G. Mattern C. Verkes R. J. Buitelaar J. Fernandez G. (2010). Testosterone Reduces amygdala-orbitofrontal cortex coupling. Psychoneuroendocrinology 35, 105–113. 10.1016/j.psyneuen.2009.09.007 19782476

[B95] Vander Weele T. J. Balboni T. A. Koh H. K. (2017). Health and spirituality. JAMA 318, 519–520. 10.1001/jama.2017.8136 28750127

[B96] Vargas K. G. Milic J. Zaciragic A. Wen K. X. Jaspers L. Nano J.. (2016). The functions of estrogen receptor beta in the female brain: a systematic review. Maturitas 93, 41–57. 10.1016/j.maturitas.2016.05.014 27338976

[B97] Veening J. G. Olivier B. (2013). Intranasal administration of oxytocin: behavioral and clinical effects, a review. Neurosci. Biobehav. Rev. 37, 1445–1465. 10.1016/j.neubiorev.2013.04.012 23648680 PMC7112651

[B98] Vegeto E. Villa A. Della Torre S. Crippa V. Rusmini P. Cristofani R.. (2020). The role of sex and sex hormones in neurodegenerative diseases. Endocr. Rev. 41, 273–319. 10.1210/endrev/bnz005 31544208 PMC7156855

[B99] Walker-Moskop R. M. (1985). “Health and cosmic continuity: hildegard of bingen's unique concerns,” in Mystics Quarterly, Vol. 11. (Penn State University Press), 19–25.

[B100] Wang Y. Liu H. Sun Z. (2017). Lamarck rises from his grave: parental environment-induced epigenetic inheritance in model organisms and humans. Biol. Rev. Camb. Philos. Soc. 92, 2084–2111. 10.1111/brv.12322 28220606

[B101] Weaver I. C. G. Cervoni N. Champagne F. A. D'Alessio A. C. Sharma S. Seckl J. R.. (2004). Epigenetic programming by maternal behavior. Nat. Neurosci. 7, 847–854. 10.1038/nn1276 15220929

[B102] Weiser M. J. Handa R. J. (2009). Estrogen impairs glucocorticoid dependent negative feedback on the hypothalamic-pituitary-adrenal axis via estrogen receptor alpha within the hypothalamus. Neuroscience 159, 883–895. 10.1016/j.neuroscience.2008.12.058 19166915 PMC5837863

[B103] Westberry J. M. Trout A. L. Wilson M. E. (2011). Epigenetic regulation of estrogen receptor beta expression in the rat cortex during aging. Neuroreport 22, 428–432. 10.1097/WNR.0b013e328346e1cf 21606911 PMC3101482

[B104] Wharton W. Gleason C. E. Olson S. R. Carlsson C. M. Asthana S. (2012). Neurobiological underpinnings of the estrogen-mood relationship. Curr. Psychiatry Rev. 8, 247–256. 10.2174/157340012800792957 23990808 PMC3753111

[B105] Wilson M. E. Westberry J. M. Prewitt A. K. (2008). Dynamic regulation of estrogen receptor-alpha gene expression in the brain: a role for promoter methylation? Front. Neuroendocrinol. 29, 375–385. 10.1016/j.yfrne.2008.03.002 18439661 PMC2460564

[B106] Yao Y. Li H. Gu Y. Davidson N. E. Zhou Q. (2010). Inhibition of SIRT1 deacetylase suppresses estrogen receptor signaling. Carcinogenesis 31, 382–387. 10.1093/carcin/bgp308 19995796 PMC2832546

[B107] Yeung E. W. Davis M. C. Ciaramitaro M. C. (2016). Cortisol profile mediates the relation between childhood neglect and pain and emotional symptoms among patients with fibromyalgia. Ann. Behav. Med. 50, 87–97. 10.1007/s12160-015-9734-z 26404060 PMC4744097

[B108] Zhang L. Hernandez V. S. (2013). Synaptic innervation to rat hippocampus by vasopressin-immuno-positive fibres from the hypothalamic supraoptic andparaventricular nuclei. Neuroscience 228, 139–162. 10.1016/j.neuroscience.2012.10.010 23085097

[B109] Zhou C. Wu Y. Ding X. Shi N. Cai Y. Pan Z. Z. (2020). SIRT1 decreases emotional pain vulnerability with associated CaMKIIα deacetylation in central amygdala. J. Neurosci. 40, 2332–2342. 10.1523/JNEUROSCI.1259-19.2020 32005763 PMC7083291

